# Sex-related differences in the association between plasma fibrinogen and non-calcified or mixed coronary atherosclerotic plaques

**DOI:** 10.1186/s13293-018-0210-x

**Published:** 2018-12-05

**Authors:** Tiewei Li, Fang Wang, Rui Peng, Shengqiang Pei, Zhihui Hou, Bin Lu, Xiangfeng Cong, Xi Chen

**Affiliations:** 1 Department of Clinical Laboratory Center, State Key Laboratory of Cardiovascular Disease, Fuwai Hospital, National Center for Cardiovascular Diseases, Chinese Academy of Medical Sciences, Peking Union Medical College, 167 Beilishi Street, Xi-Cheng District, Beijing, 100037 China; 2 Department of Radiology, Fuwai Hospital, National Center for Cardiovascular Diseases, Chinese Academy of Medical Sciences, Peking Union Medical College, Beijing, China

**Keywords:** Sex, Fibrinogen, Atherosclerosis, Non-calcified plaque, Mix plaque

## Abstract

**Background:**

Plasma fibrinogen (FIB) has been demonstrated to be a risk factor for cardiovascular disease. Patients with non-calcified plaque (NCP) or mix plaque (MP) have a higher risk of poor outcomes. However, the association between FIB and the presence of NCP or MP (NCP/MP) remains unclear, and if present, whether sex has any impact on this association remains unknown. The aim of this study was to investigate the role of FIB in predicting the presence of NCP/MP and evaluate whether sex has any impact on this association.

**Methods:**

A total of 329 subjects were recruited, and the clinical and laboratory data were collected. Plasma FIB was detected by enzyme-linked immunosorbent assay. According to whether they had coronary atherosclerotic plaques and the characteristics of the most stenotic plaque, we divided them into three groups: no plaque (NP), calcified plaque (CP), and NCP/MP.

**Results:**

Patients with NCP/MP had significantly higher FIB level in females, but not in males. Multiple logistic regression analysis showed that FIB was an independent risk factor for the presence of NCP/MP (odds ratio [OR] = 3.677, 95% CI 1.539–8.785, *P* = 0.003) in females. Receiver operating characteristic (ROC) curve analysis showed that the optimal cut-off value FIB for predicting the presence of NCP/MP was 3.41 g/L (area under curve [AUC] = 0.73, 95% CI 0.63–0.82, *P* <  0.001) in females.

**Conclusions:**

FIB is independently associated with the presence of NCP/MP in females, but not in males. These results suggest that the potential significance of FIB-lowering regimens in females with NCP/MP.

## Background

Atherosclerosis, the common cause of coronary artery disease (CAD), is characterized by the coronary atherosclerotic plaques. According to postmortem studies, plaque rupture is the cause of 75% of episodes of acute coronary syndromes (ACS), which also lead to thrombus formation. Fibrinogen (FIB), as a coagulation factor, participates in the thrombus formation.

FIB is a plasma glycoprotein, which mediates platelet aggregation and participates in the late stage of coagulation [1]. Multiple studies have demonstrated that FIB was also a pro-inflammatory factor, which played an important role in the development of atherosclerosis [2]. Several studies have shown that FIB was associated with cardiovascular disease [3, 4]. Tabakci MM et al. reported that FIB was positively correlated with the severity and complexity of coronary atherosclerosis [5]. FIB has also been shown to be an independent predictor for the adverse cardiovascular outcomes of coronary heart disease, myocardial infarction, heart failure, stroke, atrial fibrillation, and chronic obstructive pulmonary disease [4, 6–8]. Moreover, the sex differences in the clinical features have been observed. Women tend to present with coronary artery disease later in life [9], while women with acute myocardial infarction (AMI) have an unfavorable outcome compared with their male counterparts [10, 11]. In particular, previous studies showed that women had higher FIB level than men [12].

Lipid-enriched non-calcified plaque (NCP) and mixed plaque (MP) are considered as the high risk plaques due to their characteristic of being prone to rupture and their association with plaque volume progression and increased adverse outcomes [13, 14]. Hou ZH et al. reported that patients with NCP or MP (NCP/MP) had a higher risk of poor outcomes compared with those without coronary plaque or who had calcified plaques (CPs) [15]. However, there are scanty published data on the relationship between plasma FIB and the presence of NCP/MP in patients with chest pain and suspected coronary artery disease, and if the association presents, whether sex has any impact on this association remains unknown. Accordingly, the aim of this study was to investigate the role of FIB in predicting the presence of NCP/MP and to evaluate whether sex has any impact on this association.

## Methods

### Study population

From May 2015 to December 2017, we retrospectively evaluated 403 consecutive subjects who performed coronary computed tomography angiography (CCTA) due to stable typical or atypical chest pain in Fuwai Hospital (National Center for Cardiovascular Diseases, Beijing, China) and collected their complete medical history, which included clinical and laboratory data. Subjects with the following conditions were excluded from this study: (1) subjects without a lipid profiles and FIB measurements available; (2) other diseases: such as stage C or D of the progression of valvular heart disease according to the 2014 American College of Cardiology/American Heart Association guidelines for the management of patients with valvular heart disease [16], New York Heart Association (NYHA) functional class III or IV heart failure, hematological disease, cancer, and severe renal or liver diseases. Finally, a total of 329 subjects were recruited in the present study. The study protocol complied with the Declaration of Helsinki and was approved by the hospital ethics review board. Written informed consent was obtained from all the participants.

### Data acquisition

Scans were performed using a 64-row spiral CT scanner (Light Speed VCT, GE Healthcare, Milwaukee, WI, USA). According to the heart rate, patients were given 25 to 50 mg of metoprolol (Selokeen, AstraZeneca, Zoetermeer, the Netherlands) orally 1 h before scanning. Contrast medium was injected at different speed during different phases. The main scanning parameters were as follows: 64 detectors; individual detector width, 0.625 mm; gantry rotation time, 350 ms; tube voltage, 120 kV; electrocardiographically modulated tube current; pitch, 0.16 to 0.22; table feed per rotation, 400 mm; and field of view, 200 to 250 mm.

### Image analysis

The scans were retrospectively analyzed at the workstation (Deep Blue, ADW4.3, GE Healthcare, Milwaukee, WI, USA). Coronary atherosclerosis plaques were classified as CP, NCP, or MP according to their density. Briefly, lesions composed exclusively of high-density material (measured in Hounsfield Units, HU) > 130 HU were classified as CP, and ≤ 130 HU were defined as NCP. MP had the components of both CP and NCP. We grouped the participants according to the characteristic of the most stenotic coronary atherosclerotic plaque. Several studies have shown that patients with NCP/MP had a higher risk of poor outcomes [13, 14, 17]. Therefore, we combined the two groups (NCP/MP) for analysis.

### Cardiovascular risk factors assessment

The traditional risk factors for cardiovascular disease such as dyslipidemia, hypertension, diabetes mellitus, cigarette smoking, alcohol consumption, and family history of coronary heart disease (CHD) were assessed. Dyslipidemia was defined by medical history or fasting total cholesterol (TC) ≥ 5.18 mmol/L or triglyceride (TG) ≥ 1.70 mmol/L or the use of lipid-lowering medications in order to reduce lipids. Hypertension was defined as a previously established diagnosis, systolic blood pressure ≥ 140 mmHg, diastolic blood pressure ≥ 90 mmHg, or taking antihypertensive medication. Diabetes mellitus was defined as fasting glucose of ≥ 126 mg/dl, non-fasting glucose of ≥ 200 mg/dl, or receiving hypoglycemic therapy (insulin, oral hypoglycemic therapy, or dietary advice). Smoking status and alcohol consumption were ascertained by the medical history. Family history of CHD was considered as a history of CHD, myocardial infarction, coronary revascularization, or sudden cardiac death before 55 years of age for the father or 65 years of age for the mother.

### Biochemical measurements

Venous blood samples were obtained in the fasting state before undergoing CCTA from each subject and immediately detected in the department of clinical laboratory center. Serum levels of TC, TG, low-density lipoprotein cholesterol (LDL-C), high-density lipoprotein cholesterol (HDL-C), and high sensitivity C-reactive protein (hsCRP) were measured using an automatic biochemistry analyzer (Olympus Diagnostics, CA, USA) and conventional clinical analytical methods. The plasma FIB level was quantitatively measured by the method of Clauss [18] and a Stago auto analyzer with STA Fibrinogen kit (Diagnostic Stago, Taverny, France).

### Statistical analysis

Normally distributed variables were expressed as mean ± standard deviation (SD) and non-normally distributed variables were presented as median (interquartile range), which were analyzed by independent-samples *t* test, one-way ANOVA, or the Mann-Whitney *U* test, as appropriate. Categorical variables were expressed as percentages and were assessed by *χ2* or Fisher’s exact tests. Pearson or Spearman correlation test was used to examine correlations between two continuous variables when indicated. Multivariate logistic regression analysis was performed to identify the independent risk factors of the presence of NCP/MP. The risk factors were pre-specified on the basis of univariate *P* values of < 0.1. Receiver-operating characteristic (ROC) curves were used to evaluate the screening ability of FIB for the presence of NCP/MP. Youden’s index was calculated (sensitivity + specificity − 1) to determine the optimal cut-off point. All data analyses were undertaken using the statistical package SPSS 21.0 (SPSS Inc., Chicago, IL, USA). A two-sided *P* value of less than 0.05 was considered to be statistically significant.

## Results

### Baseline clinical characteristics of study subjects

A total 329 subjects undergoing CCTA were enrolled in the present study, comprised of 205 males (62.3%) and 124 females (37.7%). The participants were divided into three groups based on whether they had atherosclerotic plaques and the characteristics of the most stenotic plaque. Baseline clinical and laboratory data of the three groups were summarized in Table 1. The majority of the study population (*n* = 250, 76.0%) had atherosclerotic plaques, and the remaining 79 participants had no coronary plaques (NP). Patients with coronary atherosclerotic plaques were older and had a higher percentage of males than the patients without plaques. In addition, there was a higher proportion of participants with hypertension, diabetes, and dyslipidemia in the CP and NCP/MP groups compared with the NP group, while there was no significant in body mass index (BMI), smoking, alcohol consumption, and history of CAD among the three groups.

**Table 1:** Baseline characteristics of the study population according to the type of plaques

Variables	NP (*n* = 79)	CP (*n* = 120)	NCP/MP (*n* = 130)	*P*
Age (years)	50.1 ± 9.1	61.4 ± 9.4	58.2 ± 9.7	< 0.001
BMI (kg/m^2^)	25.1 ± 3.3	25.6 ± 3.6	25.8 ± 3.9	0.331
Male, *n* (%)	39 (49.4%)	74 (61.7%)	92 (70.8%)	0.008
Hypertension, *n* (%)	28 (35.4%)	81 (67.5%)	84 (64.6%)	< 0.001
Diabetes, *n* (%)	12 (15.2%)	29 (24.2%)	41 (31.5%)	0.029
Dyslipidemia, *n* (%)	39 (49.4%)	94 (78.3%)	114 (87.7%)	< 0.001
Smoking, *n* (%)	16 (20.3%)	31 (25.8%)	43 (33.1%)	0.117
Alcohol consumption, *n* (%)	24 (30.4%)	38 (31.7%)	42 (32.3%)	0.959
History of CAD, *n* (%)	12 (15.2%)	15 (12.5%)	17 (13.1%)	0.855
Biochemical parameters				
TC (mmol/L)	4.95 ± 0.91	4.61 ± 1.46	4.57 ± 1.29	0.092
TG (mmol/L)	1.50 (1.18, 2.19)	1.53 (1.03, 2.17)	1.57 (1.17, 2.25)	0.581
HDL-C (mmol/L)	1.20 ± 0.36	1.21 ± 0.33	1.10 ± 0.28	0.019
LDL-C (mmol/L)	3.07 ± 0.77	2.69 ± 0.88	2.78 ± 1.00	0.012
hsCRP (mg/L)	1.20 (0.65, 2.10)	1.31 (0.64, 2.80)	1.64 (0.81, 2.96)	0.070
FIB (g/L)	3.09 ± 0.69	3.06 ± 0.53	3.31 ± 0.79	0.010
Medications, *n* (%)				
Statins	30 (38.0%)	83 (69.2%)	100 (76.9%)	< 0.001
Aspirin	37 (46.8%)	95 (79.2%)	107 (82.3%)	< 0.001
Calcium antagonists	13 (16.5%)	56 (46.7%)	39 (30.0%)	< 0.001
ARB/ACEI	17 (21.5%)	49 (40.8%)	61 (46.9%)	0.001
Beta-blockers	24 (30.4%)	62 (51.7%)	70 (53.8%)	0.002
Diuretics	6 (7.6%)	28 (23.3%)	28 (21.5%)	0.013

The laboratory data showed that the level of LDL-C was lower in the CP and NCP/MP groups compared with the NP group (2.69 ± 0.88 and 2.78 ± 1.00 vs 3.07 ± 0.77 mmol/L, *P* = 0.012). The possible explanation for this observation was the higher proportion of patients using statins (69.2 and 76.9 vs 38.0%; *P* <  0.001). In addition, FIB level was significantly higher in NCP/MP group compared with the NP and CP groups. Meanwhile, the FIB level was significantly lower in men than in women (3.06 ± 0.68 vs. 3.35 ± 0.66 g/L, *P* <  0.001, data not shown).

### Changes in plasma FIB level according to sex

Furthermore, the clinical characteristics of studied population stratified by sex are presented in Table 2. In males, age, prevalence of dyslipidemia, TC, LDL-C, and medications taken (including stains, aspirin, beta-blocker and diuretics) were significantly different among the three groups, while in females, age, prevalence of hypertension and dyslipidemia, LDL-C and medications taken (including stains, aspirin, calcium antagonists and ARB/ACEI) showed significant difference among the three groups. Of note, hsCRP and FIB levels did not differ significantly in men (*P* > 0.05), whereas they did show a progressive increase among the three groups in women (*P* < 0.05).

**Table 2:** Demographic and clinical characteristics of the study subjects according to sex-specific type of plaques

Variables	Male				Female			
	NP (*n* = 39)	CP (*n* = 74)	NCP/MP (*n* = 92)	*P*	NP (*n* = 40)	CP (*n* = 46)	NCP/MP (*n* = 38)	*P*
Age (years)	48.5 ± 8.8	58.8 ± 9.8	57.5 ± 9.6	< 0.001	51.6 ± 9.3	65.5 ± 7.2	59.9 ± 9.7	< 0.001
BMI (kg/m^2^)	26.4 ± 3.0	25.7 ± 3.6	26.4 ± 4.1	0.470	23.7 ± 3.2	25.3 ± 3.6	24.5 ± 3.0	0.100
Hypertension, *n* (%)	18 (46.2%)	49 (66.2%)	60 (65.2%)	0.077	10 (25.0%)	32 (69.6%)	24 (63.2%)	< 0.001
Diabetes, *n* (%)	7 (17.9%)	20 (27.0%)	29 (31.5%)	0.280	5 (12.5%)	9 (19.6%)	12 (31.6%)	0.113
Dyslipidemia, *n* (%)	22 (56.4%)	61 (82.4%)	81 (88.0%)	< 0.001	17 (42.5%)	33 (71.7%)	33 (86.8%)	< 0.001
Smoking, *n* (%)	15 (38.5%)	29 (39.2%)	40 (43.5%)	0.803	1 (2.5%)	2 (4.3%)	3 (7.9%)	0.530
Alcohol consumption, *n* (%)	22 (56.4%)	36 (48.6%)	41 (44.6%)	0.462	2 (5.0%)	2 (4.3%)	1 (2.6%)	0.860
History of CAD, *n* (%)	6 (15.4%)	9 (12.2%)	11 (12.0%)	0.853	6 (15.0%)	6 (13.0%)	6 (15.8%)	0.934
Biochemical parameters								
TC (mmol/L)	5.03 ± 0.87	4.36 ± 1.03	4.35 ± 1.22	0.003	4.87 ± 0.96	5.00 ± 1.90	5.11 ± 1.32	0.765
TG (mmol/L)	1.83 (1.34, 2.43)	1.56 (1.09, 2.18)	1.53 (1.16, 2.14)	0.226	1.43 (0.90, 1.86)	1.49 (0.97, 2.18)	1.87 (1.20, 2.56)	0.079
HDL-C (mmol/L)	1.11 ± 0.29	1.13 ± 0.29	1.06 ± 0.27	0.328	1.28 ± 0.40	1.33 ± 0.34	1.20 ± 0.27	0.185
LDL-C (mmol/L)	3.14 ± 0.83	2.58 ± 0.87	2.65 ± 0.97	0.006	3.01 ± 0.72	2.86 ± 0.87	3.10 ± 1.00	0.441
hsCRP (mg/L)	1.45 (0.94, 1.92)	1.24 (0.60, 2.97)	1.44 (0.80, 2.90)	0.649	0.98 (0.43, 2.36)	1.50 (0.85, 2.40)	1.83 (0.89, 4.37)	0.029
FIB (g/L)	3.00 ± 0.61	2.96 ± 0.52	3.16 ± 0.80	0.161	3.18 ± 0.75	3.23 ± 0.51	3.68 ± 0.61	0.001
Medications, *n* (%)								
Statins	15 (38.5%)	53 (71.6%)	74 (80.4%)	< 0.001	15 (37.5%)	30 (65.2%)	26 (68.4%)	0.009
Aspirin	21 (53.8%)	57 (77.0%)	79 (85.9%)	< 0.001	16 (40.0%)	38 (82.6%)	28 (73.7%)	< 0.001
Calcium antagonists	9 (23.1%)	32 (43.2%)	28 (30.4%)	0.066	4 (10.0%)	24 (52.2%)	11 (28.9%)	< 0.001
ARB/ACEI	13 (33.3%)	31 (41.9%)	49 (53.3%)	0.084	4 (10.0%)	18 (39.1%)	12 (31.6%)	0.008
Beta-blockers	11 (28.2%)	42 (56.8%)	49 (53.3%)	0.010	13 (32.5%)	20 (43.5%)	21 (55.3%)	0.128
Diuretics	2 (5.1%)	21 (28.4%)	22 (23.9%)	0.015	4 (10.0%)	7 (15.2%)	6 (15.8%)	0.707

**Fig. 1:**
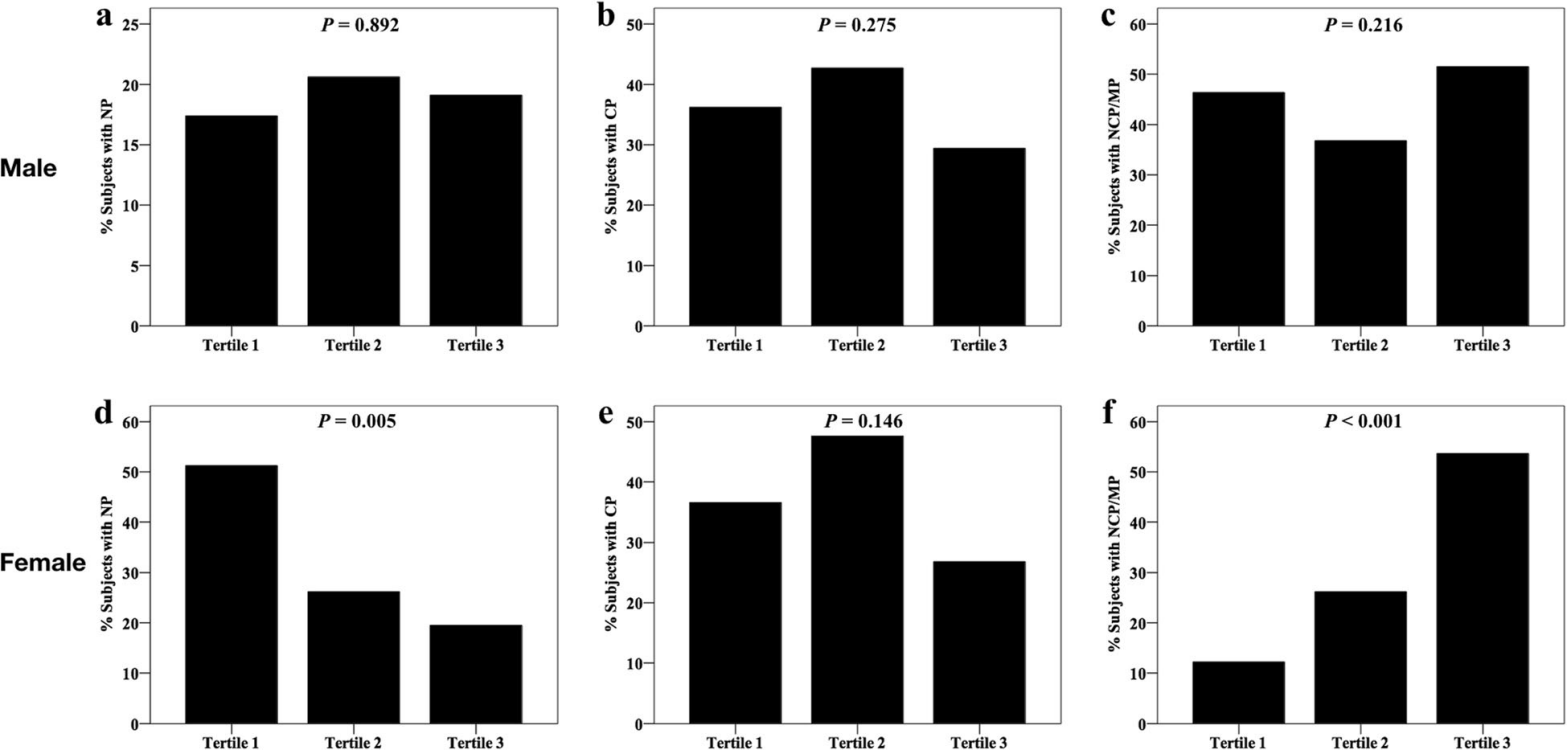
Percentage of subjects with NP, CP, and NCP/MP according to sex-specific tertiles of FIB

To further investigate the relationship between the FIB and NCP/MP, we divided the participants into three groups according to sex-specific tertiles of FIB (Table 3). Women in the third tertile were older and had higher BMI and serum hsCRP. Further analysis showed that the prevalence of hypertension and dyslipidemia showed a progressive increase among the three groups in women. However, only TG and hsCRP levels did show significant differences among the FIB tertiles in men. Of note, in females, the prevalence of NCP/MP increased significantly from 12.2% in the first tertile to 53.7% in the third tertile (*P* <  0.001) and subjects with NP were more likely to be in the first tertile (Fig. 1d–f), whereas these did not show significant difference among the FIB tertiles in males (Fig. 1a–c).

**Table 3:** Demographic and clinical characteristics of the study subjects according to sex-specific tertiles of FIB

Variables	Male				Female			
	< 2.72 (*n* = 69)	2.72–3.23 (*n* = 68)	> 3.23 (*n* = 68)	*P*	< 3.05 (*n* = 41)	3.05–3.58 (*n* = 42)	> 3.58 (*n* = 41)	*P*
Age (years)	56.4 ± 11.1	55.5 ± 9.6	56.9 ± 10.0	0.731	55.08 ± 11.8	59.6 ± 8.4	63.3 ± 9.3	0.001
BMI (kg/m^2^)	25.5 ± 2.9	26.4 ± 3.6	26.7 ± 4.5	0.140	23.6 ± 3.5	24.4 ± 3.4	25.6 ± 2.8	0.029
Hypertension, *n* (%)	38 (55.1%)	42 (61.8%)	47 (69.1%)	0.238	15 (36.6%)	21 (50.0%)	30 (73.2%)	0.004
Diabetes, *n* (%)	16 (23.2%)	21 (30.9%)	19 (27.9%)	0.594	7 (17.1%)	10 (23.8%)	9 (22.0%)	0.739
Dyslipidemia, *n* (%)	56 (81.2%)	51 (75.0%)	57 (83.8%)	0.419	23 (56.1%)	26 (61.9%)	34 (82.9%)	0.025
TC (mmol/L)	4.32 ± 1.15	4.63 ± 0.99	4.49 ± 1.20	0.272	5.03 ± 1.96	5.02 ± 1.03	4.91 ± 1.30	0.916
TG (mmol/L)	1.48 (1.00, 2.13)	1.83 (1.37, 2.49)	1.44 (1.06, 2.08)	0.012	1.46 (0.85, 2.15)	1.50 (1.05, 2.55)	1.61 (1.17, 2.16)	0.463
HDL-C (mmol/L)	1.13 ± 0.31	1.06 ± 0.27	1.09 ± 0.27	0.316	1.33 ± 0.39	1.31 ± 0.32	1.19 ± 0.32	0.132
LDL-C (mmol/L)	2.58 ± 0.95	2.81 ± 0.85	2.76 ± 0.98	0.323	2.89 ± 0.80	3.06 ± 0.80	2.99 ± 1.00	0.682
hsCRP (mg/L)	0.72 (0.47, 1.11)	1.68 (0.85, 3.04)	2.4 (1.33, 3.04)	< 0.001	0.79 (0.40, 1.51)	1.12 (0.74, 1.85)	3.01 (1.66, 5.16)	< 0.001
CCTA data, *n* (%)								
NP	12 (17.4%)	14 (20.6%)	13 (19.1%)	0.892	21 (51.2%)	11 (26.2%)	8 (19.5%)	0.005
CP	25 (36.3%)	29 (42.6%)	20 (29.4%)	0.275	15 (36.6%)	20 (47.6%)	11 (26.8%)	0.146
NCP/MP	32 (46.4%)	25 (36.8%)	35 (51.5%)	0.216	5 (12.2%)	11 (26.2%)	22 (53.7%)	< 0.001

### Sex-specific correlation between FIB and other cardiovascular risk factors

To explore the correlation between variables and FIB, Spearman correlation analysis was performed in the study (Table 4). In women, FIB was positively correlated with age (*r* = 0.295, *P* = 0.001), BMI (*r* = 0.177, *P* = 0.049), and hsCRP (*r* = 0.521, *P* <  0.001), while FIB was only significantly correlated with hsCRP (*r* = 0.580, *P* <  0.001) in men. In addition, there was no significant correlation between FIB and TC (*r* = 0.580, *P* <  0.001), TG, HDL-C, and LDL-C in both sexes.

**Table 4:** Correlation between FIB and other cardiovascular risk factors

Variables	Male (*n* = 205)		Female (*n* = 124)	
	*r*	*P*	*r*	*P*
Age (years)	0.058	0.412	0.295	0.001
BMI (kg/m^2^)	0.123	0.079	0.177	0.049
TC (mmol/L)	0.025	0.718	0.052	0.568
TG (mmol/L)	0.022	0.758	0.109	0.226
HDL-C (mmol/L)	− 0.034	0.631	− 0.148	0.100
LDL-C (mmol/L)	0.030	0.671	0.064	0.477
hsCRP (mg/L)	0.580	< 0.001	0.521	< 0.001

### Multivariable binary logistic regression

Multivariable binary logistic regression analysis was performed to identify the independent predictors of the presence of NCP/MP in females. Variables with *P* <  0.1 in univariate analysis were included in the model of multivariate analysis including diabetes, dyslipidemia, usage of statin, usage of beta-blocker, hsCRP, and FIB. As shown in Table 5, multivariate analysis showed that FIB was the independent predictor of the presence of NCP/MP (odds ratio [OR] = 3.677, 95% CI 1.539–8.785, *P* = 0.003). Furthermore, the highest FIB tertile was also independently associated with the presence of NCP/MP.

**Table 5:** Regression analysis to assess the presence of NCP/MP according to FIB

Model	Variables	Men		Women	
		OR (95% CI)	*P*	OR (95% CI)	*P*
Model 1	FIB	1.491 (0.980–2.269)	0.062	3.206 (1.625–6.326)	0.001
	FIB tertiles				
	Tertile 1	1		1	
	Tertile 2	0.672 (0.339–1.331)	0.255	2.555 (0.800–8.159)	0.113
	Tertile 3	1.226 (0.627–2.399)	0.551	8.337 (2.723–25.521)	< 0.001
Model 2	FIB	1.416 (0.854–2.347)	0.178	2.557 (1.164–5.617)	0.019
	FIB tertiles				
	Tertile 1	1		1	
	Tertile 2	0.626 (0.304–1.288)	0.203	2.386 (0.723–7.878)	0.153
	Tertile 3	0.986 (0.463–2.101)	0.970	5.823 (1.696–19.994)	0.005
Model 3	FIB	1.416 (0.843–2.369)	0.189	3.677 (1.539–8.785)	0.003
	FIB tertiles				
	Tertile 1	1		1	
	Tertile 2	0.654 (0.312–1.370)	0.260	2.885 (0.797–10.439)	0.106
	Tertile 3	1.004 (0.465–2.169)	0.992	8.531 (2.153–33.802)	0.002

*Model 1* unadjusted

*Model 2* adjusted for dyslipidemia, hsCRP (*P* < 0.05 in univariate analysis)

*Model 3* adjusted for dyslipidemia, diabetes, statins, beta-blocker and hsCRP (*P* < 0.1 in univariate analysis)

### ROC curve analysis

**Fig. 2:**
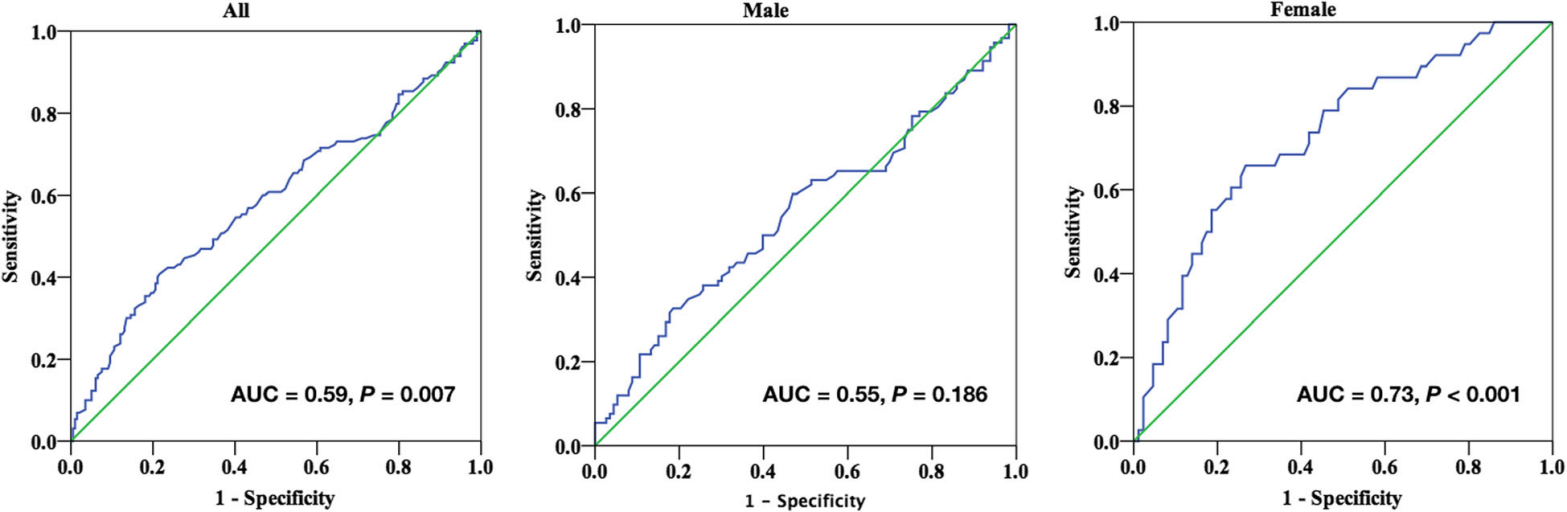
ROC curves for NCP/MP in all population, males, and females

**Fig. 3:**
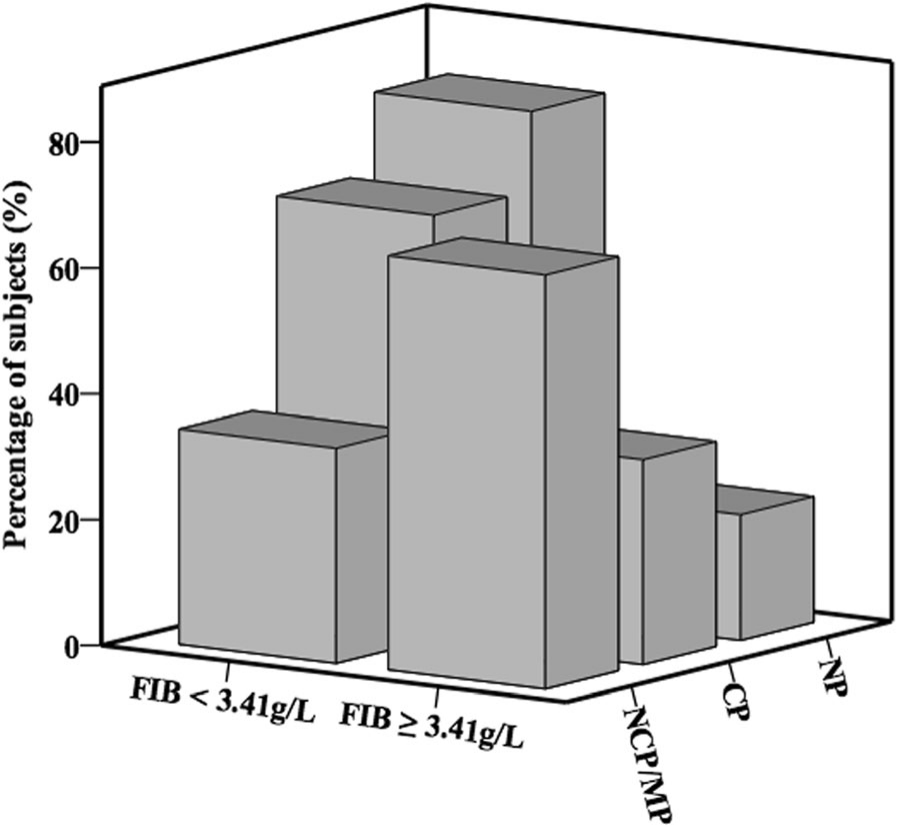
Percentage of subjects with NP, CP, and NCP/MP according to the level of FIB in females

ROC curve analysis was performed to evaluate the utility of FIB in predicting the presence of NCP/MP in women. Area under the ROC curve (AUC) indicated a well discriminatory power of FIB (AUC = 0.73, 95% CI 0.63–0.82, *P* <  0.001; Fig. 2) in females. Optimal cut-off value of FIB for predicting the presence of NCP/MP was 3.41 g/L, with a sensitivity of 66% and specificity of 73%. According to this cut-off value, we divided the female subjects into two groups (high FIB group ≥ 3.41 g/L and low FIB group < 3.41 g/L). As shown in Fig. 3, the high FIB group tended to have higher prevalence of NCP/MP and lower prevalence of NP compared to the low FIB group (*P* <  0.05).

## Discussion

Compared with CP, NCP was more prone to be in the culprit lesions in patients with ACS and patients with NCP had a higher risk of future ACS events [13, 17, 19]. Moreover, Pundziute et al. found that thin cap fibroatheroma was most frequently observed in MP on MSCT and the number of MP determined by multislice computed tomography was an independent predictor of acute cardiac events [14]. In addition, patients with NCP/MP had a three times higher risk of 3-year major adverse cardiac events in outpatients with suspected CAD [15]. So, NCP and MP in CCTA could be considered as high-risk plaques. However, owing to the radiation exposure, complicated procedure, and high cost, CCTA is not suitable for large-scale screening to identify NCP/MP. Therefore, screening circulating biomarker for early identification of the presence of NCP/MP might provide valuable information for preventing the future poor outcomes.

FIB is a soluble 340 kDa glycoprotein synthesized in hepatocytes that circulates in plasma, which plays a vital role in blood clotting [1, 20]. FIB also has a pro-inflammatory role on the different vascular cells implicated in atherogenesis [21]. FIB has been shown to activate pro-inflammatory pathways, which results in local production of inflammatory cytokines, such as MCP-1, TNF-α, IL-8 and endothelin-1 in endothelial cells [22], vascular smooth muscle cells [23], monocytes/macrophages [24, 25], and neutrophils [26]. Moreover, FIB participated in the formation and development of atherosclerosis lesion through increasing blood viscosity, stimulating platelet aggregation [2, 20], injuring endothelial cells [27, 28], and promoting migration and proliferation of vascular smooth muscle cells (VSMCs) [29]. Numerous epidemiological investigations as well as meta-analysis studies suggested that it was a powerful predictor for cardiovascular disease, including CHD, myocardial infarction, stroke, and other vascular mortality [4, 30–32]. In addition, under Adult Treatment Panel III guidelines, additional targeted assessment of the FIB in people at intermediate risk for a cardiovascular event could help prevent one additional event over the course of 10 years [3]. Immunohistochemical studies showed that FIB was the component of all stable and unstable coronary atherosclerotic plaques, while FIB showed a significant predominance in unstable angina. Clinical studies have demonstrated that plasma FIB level was associated with coronary plaque morphology [33], plaque burden [34], and the plaque progression [35]. Moreover, recent evidence showed that the FIB degradation products (FDP) were independently associated with larger plaques and greater plaque necrotic core [36]. However, there are few published data on the relationship between the FIB and the presence of NCP/MP.

In the present study, we found that NCP/MP group had the highest level of FIB among the three groups in the overall study population. However, after stratified by sex, the statistical significance of FIB among three groups only exited in women, not in men. Multivariate analysis showed that FIB was the independent predictor of the presence of NCP/MP in women. Moreover, ROC curve analysis showed that plasma FIB had a well discriminatory utility in predicting the presence of NCP/MP in females.

Of note, these associations were found only in females indicating that sex might have some impact on the relationship between FIB level and the presence of NCP/MP. Consistent with our data, several studies have shown that sex had effects on the plasma FIB level, in which there was a higher level of FIB in women than that in men [12, 37]. In patients with obstructive sleep apnea, the correlation between FIB and the sleep data were more apparent in female patients than in males [12], which was similar to our results that the association between FIB and the presence of NCP/MP only exited in females. In addition, there also appeared to be significant difference in plaque morphology in men and women. Epidemiological studies have shown that eroded plaques were more frequent in women and the most common cause of sudden death in women was acute thrombus upon plaque erosion, while men had greater necrotic lipid core and hemorrhagic area of the plaque compared with women [38, 39]. This phenomenon of sex-related differences in atherosclerosis may be due to the different role of estrogens and androgens in atherosclerosis. Androgens could promote the foam cell formation, the expression of atherogenic genes and vascular endothelial cell apoptosis, which facilitated the development of atherosclerosis, while estrogen had anti atherosclerotic effects through promoting the NO synthesis, vasodilation and hyaluronan deposition, and inhibiting oxidative stress and the proliferation of VSMC [40]. Meanwhile, several studies demonstrated that testosterone has an inverse association with FIB level [41–43], while estradiol and estrone were positively associated with plasma FIB level in post-menopausal women [44, 45]. These phenomena of sex-related differences in atherosclerosis and FIB level may partly be the reason for the sex-related differences in the association between plasma FIB and the presence of NCP/MP.

Of course, there are several limitations in our study. First, the sample size of the study was relatively small. The findings of this study need further validation in a large population. Second, this was a cross-sectional study, which did not permit the determination of causality. Third, serial measurements of FIB and changes of the characteristic of plaque might provide more implications and be useful to further explore the dynamic correlation between them. Finally, all of the enrolled subjects were Chinese and had symptoms of chest pain. The findings of this study cannot be applicable for other ethnic groups and general population.

## Conclusions

To the best of our knowledge, this is the first study to investigate the sex-related differences in the relationship between FIB and the presence of NCP/MP. As expected, FIB level was higher in females than in males and showed a progressive increase among the three groups only in females. Moreover, our multivariate analysis showed that FIB was independently associated with the presence of NCP/MP and ROC curve indicated a well discriminatory power of plasma FIB in predicting the presence of NCP/MP in females. These findings suggest the sex-related differences should be taken into account in therapeutic approaches to regress NCP/MP by using FIB-lowering drugs.

## Abbreviations

ACEI: Angiotensin-converting enzyme inhibitors
ARB: Angiotensin II receptor blockers
AUC: Area under curve
BMI: Body mass index
CAD: Coronary artery disease
CCTA: Coronary computed tomography angiography
CI: Confidence interval
CP: Calcified plaque
FIB: Fibrinogen
HDL-C: High-density lipoprotein-cholesterol
hsCRP: High sensitivity C-reactive protein
LDL-C: Low-density lipoprotein-cholesterol
MP: Mixed plaque
NCP: Non-calcified plaque
NP: No plaque
OR: Odds ratio
ROC: Receiver operating characteristic
TC: Total cholesterol
TG: Triglycerides
